# Hashimoto’s thyroiditis induces neuroinflammation and emotional alterations in euthyroid mice

**DOI:** 10.1186/s12974-018-1341-z

**Published:** 2018-10-29

**Authors:** Yao-Jun Cai, Fen Wang, Zhang-Xiang Chen, Li Li, Hua Fan, Zhang-Bi Wu, Jin-Fang Ge, Wen Hu, Qu-Nan Wang, De-Fa Zhu

**Affiliations:** 1 Department of Endocrinology, Anhui Geriatric Institute, the First Affiliated Hospital of Anhui Medical University, Hefei, 230032 China; 2 Anhui Key Laboratory of Bioactivity of Natural Products, School of Pharmacy, Anhui Medical University, Hefei, 230032 China; 3 Department of Pathology, Anhui Provincial Hospital Affiliated to Anhui Medical University, Hefei, 230032 China; 4 Department of Toxicology, School of Public Health, Anhui Medical University, Hefei, 230032 China

**Keywords:** Hashimoto’s thyroiditis, Neuroinflammation, Anxiety, Depression, Serotonin

## Abstract

**Background:**

Although studies have reported an increased risk for mood disorders in Hashimoto’s thyroiditis (HT) patients even in the euthyroid state, the mechanisms involved remain unclear. Neuroinflammation may play a key role in the etiology of mood disorders in humans and behavioral disturbances in rodents. Therefore, this study established a euthyroid HT model in mice and investigated whether HT itself was capable of triggering neuroinflammation accompanied by emotional alterations.

**Methods:**

Experimental HT was induced by immunizing NOD mice with thyroglobulin and adjuvant twice. Four weeks after the last challenge, mice were tested for anxiety-like behavior in the open field and elevated plus maze tests and depression-like behavior in the forced swimming and tail suspension tests. Then, animals were sacrificed for thyroid-related parameter measure as well as detection of cellular and molecular events associated with neuroinflammation. The changes in components of central serotonin signaling were also investigated.

**Results:**

HT mice showed intrathyroidal monocyte infiltration and rising serum thyroid autoantibody levels accompanied by normal thyroid function, which defines euthyroid HT in humans. These mice displayed more anxiety- and depressive-like behaviors than controls. HT mice further showed microglia and astrocyte activation in the frontal cortex detected by immunohistochemistry, real-time RT-PCR, and transmission electron microscopy (TEM). These observations were also accompanied by enhanced gene expression of proinflammatory cytokines *IL-1β* and *TNF-α* in the frontal cortex. Despite this inflammatory response, no signs of neuronal apoptosis were visible by the TUNEL staining and TEM in the frontal cortex of HT mice. Additionally, IDO1 and SERT, key serotonin-system-related genes activated by proinflammatory cytokines, were upregulated in HT mice, accompanied by reduced frontal cortex serotonin levels.

**Conclusions:**

Our results are the first to suggest that HT induces neuroinflammation and alters related serotonin signaling in the euthyroid state, which may underlie the deleterious effects of HT itself on emotional function.

## Background

Hashimoto’s thyroiditis (HT) is a frequent autoimmune thyroid disease, affecting approximately 5% of the general population, especially women [1]. HT is characterized by intrathyroidal monocyte infiltration along with rising serum autoantibodies, such as anti-thyroglobulin antibody (anti-Tg) and anti-thyroid peroxidase antibody (anti-TPO), and is the leading cause of hypothyroidism worldwide [2]. However, most patients (approximately 79.3%) show normal thyroid function at diagnosis [3] and may be euthyroid for many years [2]. Hypothyroidism may lead to neuropsychological deficits, including depression and anxiety [4]. Only in recent years, a high prevalence of psychiatric involvement in HT patients has been increasingly recognized, independent of thyroid function [5–9]. Symptoms of depression and anxiety are more common in euthyroid HT patients than in the general population, with up to 52.9% demonstrating affective illness [7]. Neuroimaging data of euthyroid HT patients, even those without psychiatric symptoms, reveals cerebral perfusion impairments, particularly in the frontal cortex [10], a key brain region for the control of emotional behaviors [11]. In line with this, another imaging study showed decreased gray matter density in the left inferior frontal gyrus in these patients [12]. In addition, a severe neuropsychiatric disorder independent of thyroid status has been described to exceptionally target patients suffering with HT [13, 14]. Taken together, these data strongly suggest a primary brain-specific mechanism that is independent of thyroid hormone level. However, the mechanisms of brain injury responsible for the psychological impairments in the context of euthyroid HT remain unclear.

HT is the most prevalent autoimmune disease, frequently co-occurring with other autoimmunological diseases [15], including rheumatoid arthritis and systemic lupus erythematosus. These illness have also been found to present a high comorbidity with depression and anxiety [16, 17], the mechanism of which has been reported to be associated with significant neuroinflammation [17, 18]. Neuroinflammation is an essential innate response against brain injury. However, uncontrolled neuroinflammation can result in a series of deleterious consequences involving brain cells, immune cells, and signaling molecules [19]. Glial cells, including microglia and astroglia, are the immune cells of the central nervous system and the main cellular regulators of neuroinflammation [20]. Normally, glial cells exist in the resting state, but under pathological conditions, they become over-activated and release a plethora of neurotoxic species, such as proinflammatory cytokine interleukin-1β (IL-1β), tumor necrosis factor-α (TNF-α), and interleukin-6 (IL-6) [20]. These events appear to negatively impact the synthesis and reuptake of neurotransmitters relevant to mood regulation, especially serotonin (5-HT) [21]. As such, neuroinflammation, characterized by neuroglia activation and the related generation of pro-inflammatory cytokines, has been acknowledged as a triggering factor for psychiatric conditions [22, 23]. Given the significant role of neuroinflammation in mental pathology, we hypothesize that neuroinflammation may provide a mechanism for the pathogenesis of psychological impairments in the context of euthyroid HT.

To test this hypothesis, this study built a classical model for the human disease HT in which female NOD mice were immunized with thyroglobulin [24] and investigated whether HT itself was capable of triggering neuroinflammation accompanied by anxiety/depressive-like behaviors in mice. As the frontal cortex is reported to be predominantly affected in euthyroid patients with HT [10, 12] and play a substantial role in mood regulation [11], we focused on the frontal cortex and set out to examine the cellular and molecular events associated with neuroinflammation, such as the activation status of microglia and astrocytes as well as the expression of proinflammatory cytokines IL-1β, TNF-α, and IL-6. The consequences of such inflammation on the neurons and the components of serotonin signaling were also examined.

## Methods

### Animals

Female NOD mice (8–9 weeks old; 22~25 g) were obtained from Beijing HFK Bioscience Co., Ltd. (Beijing, China) and randomly assigned into two groups: the control group (Con group, *n* = 10) and Hashimoto’s thyroiditis group (HT group, *n* = 10). These mice were housed in cohorts of three to four under standard laboratory conditions (23 ± 2 °C, a 12-h light/12-h dark cycle, 55 ± 5% humidity) and had ad libitum access to tap water and rodent chow. The NOD mouse strain is a common model of autoimmune diseases. The animals spontaneously develop autoimmune diabetes with aging. Considerable evidence has confirmed that diabetes in NOD mice can be inhibited by a single injection of complete Freund’s adjuvant [25–27], so this condition may not be a major issue when NOD mice, following immunization with thyroglobulin in complete Freund’s adjuvant, are used for HT in this study [24].

### Immunization and experimental design

**Fig. 1:**
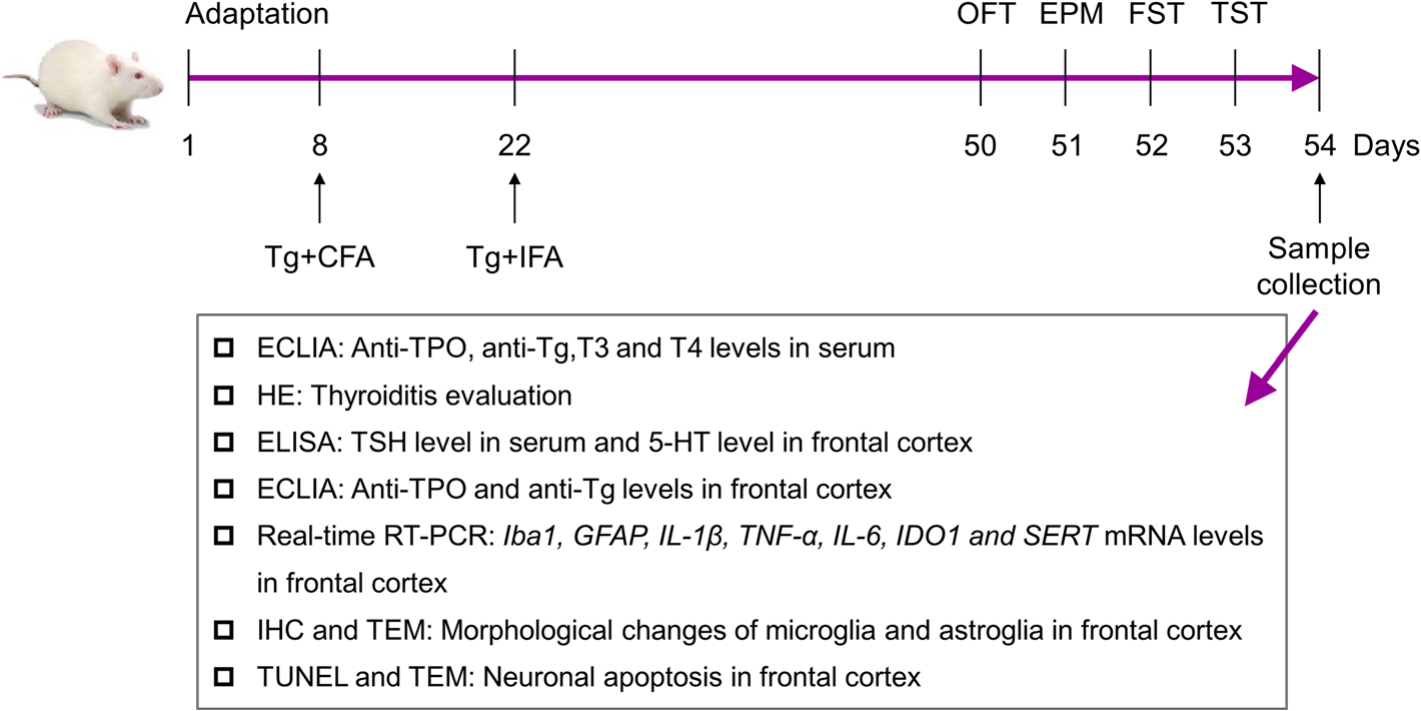
Experiment schedule. Annotations: Tg, thyroglobulin; CFA, complete Freund’s adjuvant; IFA, incomplete Freund’s adjuvant; OFT, open field test; EPM, elevated plus maze; FST, forced swimming test; TST, tail suspension test; Anti-TPO: anti-thyroid peroxidase antibody; Anti-Tg: anti-thyroglobulin antibody; T3: triiodothyronine; T4: thyroxin; TSH: thyroid-stimulating hormone; 5-HT: serotonin; Iba1: ionized calcium-binding adapter molecule 1; GFAP: glial fibrillary acidic protein; IL-1β: interleukin-1β; TNF-α: tumor necrosis factor-α; IL-6: interleukin-6; IDO1: indoleamine-2,3-dioxygenase; SERT: serotonin transporter

After 7 days of acclimatizing, mice in the HT group were challenged with 25 μg porcine thyroglobulin (Tg; Sigma-Aldrich, USA) emulsified in complete Freund’s adjuvant (CFA; Sigma-Aldrich, USA) injected subcutaneously at the base of the tail, and with a booster injection of an equal dose of Tg in incomplete Freund’s adjuvant (IFA; Sigma-Aldrich, USA) performed 14 days later. Mice treated with phosphate buffered saline (PBS) instead of Tg at the same time served as controls. Four weeks after the second challenge, the behavioral and biochemical parameters of all mice were evaluated (please see Fig. 1).

### Behavioral tests

Behavioral tests were performed to examine the anxiety/depression-like states in animals. Mice were taken to the test room 60 min before the test. Behavioral procedures were conducted between 0830 and 1200 h in a dim and quiet room. The observers were blind to the experimental design. During the test, all animals were tracked and recorded by ANY-Maze™ Video Tracking Software (Stoelting Co., Illinois, USA) with a digital camera.

### Open field test (OFT)

The OFT is widely applied to test motor and anxiety-like behavior of rodents in a novel environment [28, 29]. The equipment consisted of a black square arena (40 × 40 cm) with walls of 40 cm in height. The computer defined the grid lines dividing the box floor into 16 equal-sized squares, with the central four squares regarded as the center. Each mouse was gently placed at one corner of the arena facing the wall and videotaped for 5 min. Time in the center, entries into the center, total moving distance, mean speed, rearing number, and grooming number were analyzed. After each test, the equipment was wiped down with ethanol.

### Elevated plus maze (EPM)

The EPM is a well-validated instrument used to analyze anxiety-like traits in rodents [28]. The maze (80 cm in height from the ground) is made up of two opposite open arms (30 cm × 5 cm, without edges), two opposite closed arms (30 cm × 5 cm × 15 cm), and a central zone (5 cm × 5 cm). Each animal was gently mildly placed in the central area facing an open arm and left to move freely for 5 min. Open-arm entries, open-arm time, the percentage of open/total-arm entries, and the percentage of open/total-arm time were recorded and analyzed. After each test, the equipment was wiped down with ethanol.

### Forced swimming test (FST)

The FST was applied to measure depressive-like behavior based on previously described methods [30]. Each mouse was gently placed into a plexiglass cylinder (30 cm in height, 11 cm in diameter) filled with 25 cm of tap water (24 ± 1 °C) for a 6-min test. The trial was videotaped for later manual summation of immobility time during the final 4 min by using a stopwatch. Immobility, defined as floating with only the small movements required to keep the head above water, was evaluated by two observers blind to experimental design and averaged. After each trial, animals were towel-dried, placed under a heat lamp, and returned to the housing cage. The water was replaced in between mice.

### Tail suspension test (TST)

The TST was applied to measure depressive-like behavior as described by Ge et al [29]. In brief, each animal was hung by adhesive tape and suspended from a fixed hook (50 cm in height from the floor) for 6 min. The trial was videotaped for later manual summation of immobility time during the final 4 min by using a stopwatch. Immobility, defined as no active movements except for respiration and whisker movement, was evaluated by two observers blind to experimental design and averaged. After each test, the apparatus was cleaned.

### Tissue preparation

On the last day following the TST, mice were deeply anesthetized and randomly sacrificed in the morning (09:00–11:30 am). Blood, the thyroid, and the brain were collected immediately. Blood samples were centrifuged for measuring serological parameters. The thyroids were collected for histopathology. The brains was carefully dissected on ice and randomly assigned for later assay: right frontal lobes for electrochemiluminescence immunoassay, enzyme-linked immunosorbent assay, and transmission electron microscopy and left frontal lobes for immunohistochemistry, TUNEL staining, and real-time RT-PCR.

### Electrochemiluminescence immunoassay (ECLIA)

Serum samples were kept at − 80 °C until use. The levels of serum triiodothyronine (T3), thyroxin (T4), anti-TPO, and anti-Tg were quantified through ECLIA on Cobas e411 immunoassay analyzer (Roche, Mannheim, Germany). The procedures for ECLIA were as described in detail elsewhere [31]. In addition, the frontal cortex tissues were homogenized in PBS at a ratio of 1:9 (weight to volume) and then centrifuged at 12,000*g* for 20 min at 4 °C. The supernatants were collected for an analysis of protein content using the BCA method and then for 5-HT content measure as well as detection of thyroid autoantibody levels. Frontal cortex anti-TPO and anti-Tg levels in mice were measured with ECLIA and modified by the corresponding protein concentration. Data were expressed as international units per milligram protein of brain issue.

### Enzyme-linked immunosorbent assay (ELISA)

The levels of thyroid-stimulating hormone (TSH) in serum and 5-HT in the frontal cortex were analyzed utilizing ELISA kits (TSH: USCN Life Science Inc., Wuhan, China; 5-HT: Enzo Life Science, NY, USA) [32, 33], in accordance with the manufacturer’s guidelines. Data were expressed as picograms per milliliter of serum or picograms per milligram protein of brain issue.

### Hematoxylin and eosin (HE)

For histopathology, fresh thyroid tissues were fixed and processed for HE. From each animal, five noncontiguous coronal sections were used to examine thyroid histopathology. The thyroiditis classification standard was based on the percentage of thyroid infiltrated, as previously described [24]: 0 = absence of infiltrate; 1 = interstitial accumulation of inflammatory cells around one or two follicles; 2 = one or two foci of inflammatory cells reaching the size of a follicle; 3 = 10–40% inflammatory cells infiltration; 4 = greater than 40% inflammatory cells infiltration. The histological scores were evaluated and averaged by two investigators blind to experimental design.

### Immunohistochemistry (IHC)

The fixed left hemispheres were processed for paraffin embedding. Serial coronal sections (5 μm thick) were cut at the levels of the frontal cortex following a mouse brain atlas [34]. Histological sections were used to immunostain ionized calcium-binding adapter molecule 1 (Iba1, Wako) and glial fibrillary acidic protein (GFAP, Abcam). Iba1 immunolabeling was able to identify activated microglia characterized by enlarged, darkened soma and thickened processes [35]. GFAP immunolabeling allowed for identification of activated astrocytes as demonstrated by hypertrophy of the cell body and stem processes [36]. For immunohistochemical analysis, five sections (1/5 serial sections) per antibody were used. Sections were dewaxed in xylene and rehydrated through gradient ethanol. After optimum antigen retrieval and quenching endogenous peroxidase, sections were incubated overnight at 4 °C with different primary antibodies, such as rabbit anti-Iba1 (1:500) or rabbit anti-GFAP (1:2000). Subsequently, appropriate secondary antibodies were used, followed by incubation with avidin-biotin complex (Zsgb-bio, Beijing, China). Finally, chromogen was added to each section and counterstained with hematoxylin.

For histopathological analysis, slides were photographed with a Nikon 80i microscope (Nikon, Tokyo, Japan). In each section, three nonoverlapping fields were randomly selected in the frontal cortex at magnification × 100. The numbers of activated microglia and astroglia in the frontal cortex were calculated [37, 38] . In addition to cell counting, Iba1 and GFAP immunoreactivity was analyzed by determining the percentage of Iba1 or GFAP-stained area, respectively, as described previously [38, 39]. All images were analyzed by two blinded observers using the public domain NIH ImageJ Program.

### Transmission electron microscopy (TEM)

For ultrastructure studies of microglia, astrocytes, and neurons, the frontal cortex in HT mice and the equivalent area in Con mice were obtained according to the mouse brain atlas. The entire TEM procedure was well described in our previous study [40]. Observations of neuroglia and neurons were made using magnifications ranging from 8000 to 12,000×.

### Terminal deoxynucleotidyl transferase-mediated dUTP-biotin nick end labeling (TUNEL) staining

Apoptotic neurons in the frontal cortex were visualized using a TUNEL kit (Roche, Basel, Switzerland) following the manufacturer’s protocol. Briefly, serial coronal sections from the left frontal cortex were dewaxed in xylene and rehydrated through gradient ethanol. After rinsing with PBS, the sections were incubated with proteinase K solution at 37 °C for 20 min. Then they were incubated at 37 °C for 2 h with TUNEL reaction mixture in a dark, humidified chamber. Following five PBS washes, the nuclei were stained with DAPI (Servicebio, Wuhan, China) at room temperature for 10 min and slides were coverslipped. TUNEL-positive signals in the frontal cortex were examined using a fluorescent microscope (Nikon Eclipse C1).

### RNA purification and real-time RT-PCR

Total RNA was extracted from the frontal cortex using TRI reagent (Invitrogen, Carlsbad, CA) and treated with RNase-free DNase followed by reverse transcription with AMV (Promega, Wisconsin, USA) according to the manufacturer’s guidelines. The primers used for PCR are listed as follows: *Iba1*: Forward Primer (FP) - CTT GAA GCG AAT GCT GGA GAA, Reverse Primer (RP) - GGC AGC TCG GAG ATA GCT TT; *GFAP*: FP - CGG AGA CGC ATC ACC TCTG, RP - TGG AGG AGT CAT TCG AGA CAA; *IL-1β*: FP - GAA ATG CCC CTT TTG ACA GTG, RP - TGG ATG CTC TCA TCA GGA CAG; *TNF-α*: FP - CAG GCG GTG CCT ATG TCTC, RP - CGA TCA CCC CGA AGT TCA GTAG; *IL-6*: FP - CTG CAA GAG ACT TCC ATC CAG, RP - AGT GGT ATA GAC AGG TCT GTT GG; *IDO1*: FP - TGG CGT ATG TGT GGA ACCG, RP - CTC GCA GTA GGG AAC AGC AA; *SERT*: FP - CTC CGC AGT TCC CAG TAC AAG, RP - CAC GGC ATA GCC AAT GAC AGA; *18S*: FP - GTA ACC CGT TGA ACC CCA TT, RP - CCA TCC AAT CGG TAG TAG CG. The PCR was conducted on a Light Cycler® 480 Instrument (Roche, Mannheim, Germany) with an initial hold step (95 °C for 5 min) and 50 cycles of 15 s at 95 °C, 15 s at 60 °C, and 30 s at 72 °C. Relative mRNA expression was analyzed using the 2^−ΔΔCt^ method and normalized to the 18s rRNA levels.

### Statistical analysis

All statistical analyses were performed using GraphPad Prism 5.0 (San Diego, CA, USA). Values are presented as the mean ± standard error (SEM). Mann-Whitney *U* test was used to analyze the severity of thyroiditis. The other data were compared by unpaired two-tailed Student’s *t* test. The significance criterion was set at *p* value < 0.05.

## Results

### Building a euthyroid HT model in mice

**Fig. 2:**
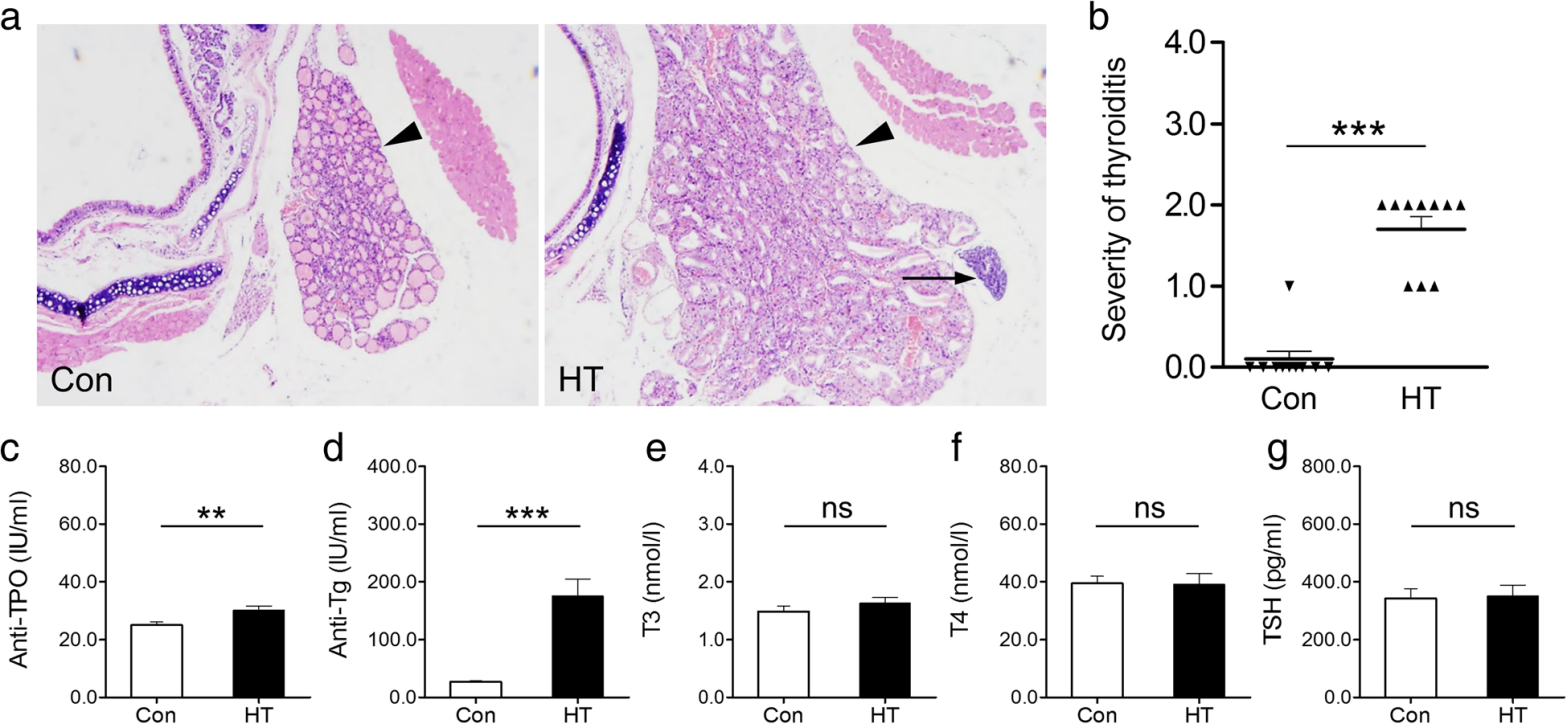
Building a euthyroid HT model in mice. **a** Representative thyroid sections stained with HE shown at magnification × 50. Con mice displayed a normal thyroid gland. HT mice manifested thyroid enlargement with destroyed thyroid follicles and prominent monocyte infiltration. Arrowhead: thyroid gland; Arrow: infiltrated monocytes. **b** Quantitation of the degree of monocyte infiltration in thyroids. The analysis was done as described in the Methods. **c**–**g** Serum levels of thyroiditis-related parameters, including anti-TPO (**c**), anti-Tg (**d**), T3 (**e**), T4 (**f**), and TSH (**g**). Data are presented as the mean ± SEM, *n* = 10; ns, no statistical significance; ^**^p < 0.01, and ^***^p < 0.001, vs. Con

**Fig. 3:**
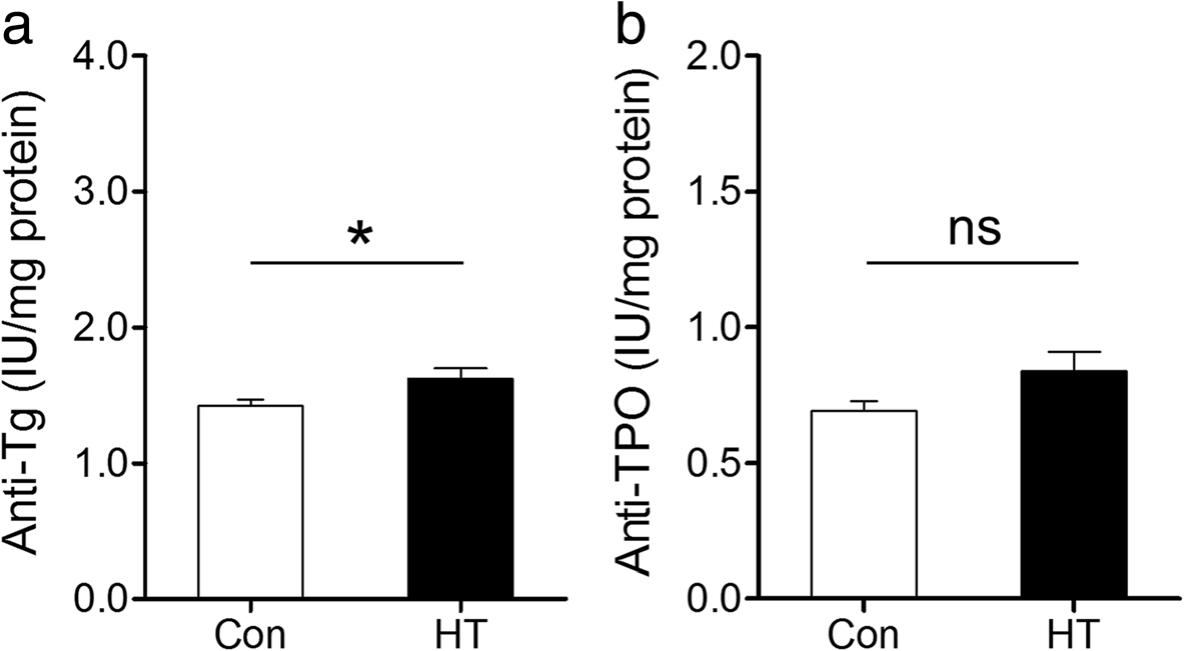
Frontal cortex levels of anti-Tg and anti-TPO in mice. ECLIA was performed to detect thyroid autoantibody levels in mouse brain homogenate supernatant. **a** Frontal cortex anti-Tg levels. **b** Frontal cortex anti-TPO levels. Data are presented as the mean ± SEM, *n* = 7; ns, no statistical significance; ^*^p < 0.05, vs. Con

As depicted in Fig. 2a, histological examination showed that control mice had intact thyroid follicles with an even distribution, and monocyte infiltration was hardly found in thyroid tissues. In contrast, HT mice displayed significant thyroid enlargement with disorderly and destroyed thyroid follicles, and monocyte infiltration was found more or less in thyroid tissues. Further quantitative analysis revealed that the severity of thyroiditis in HT mice was significantly higher than that in controls (Fig. 2b). On the other hand, as seen in Fig. 2c–g, serum concentrations of thyroid autoantibodies (anti-TPO and anti-Tg) in the HT group were apparently higher than those in the Con group (Fig. 2c, d), while no differences in serum T3, T4, or TSH levels (Fig. 2e–g) was detected between groups. Moreover, frontal cortex levels of anti-Tg were significantly higher in HT mice than those in controls (Fig. 3a). As well, there was a similar, albeit not significant, tendency for frontal cortex anti-TPO levels between groups (*p* = 0.09, Fig. 3b). Taken together, these findings indicated that a euthyroid HT model was successfully established in mice. Since NOD mice spontaneously develop autoimmune diabetes, we examined serum glucose levels in all mice using a glucose analyzer (Roche, Indianapolis, IN), and no significant difference was detected in serum glucose levels between Con mice and HT mice (5.50 ± 0.55 vs. 6.31 ± 0.52 mmol/L, *p* = 0.30, *n* = 10), in line with previous studies [24, 25].

### Euthyroid HT induces anxiety-like behavior in mice

**Fig. 4:**
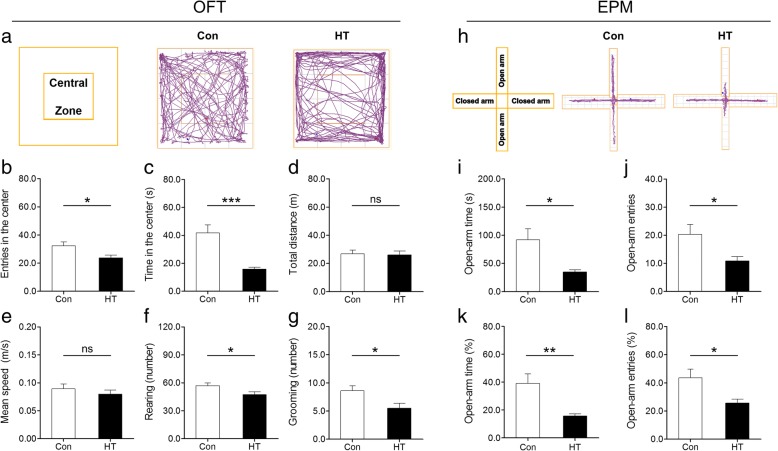
Euthyroid HT induces anxiety-like behavior in mice. The OFT and EPM were used to evaluate anxiety-like states in animals. **a**–**g** Behavioral performances in the OFT, including representative path tracings during the OFT (**a**), entries into the center (**b**), time spent in the center (**c**), total distance (**d**), mean speed (**e**), number of rearings (**f**) number of groomings (**g**). **h-l** Behavioral performances in the EPM, including representative path tracings during the EPM (**h**), open-arm time (**i**), open-arm entries (**j**), percentage of open/total-arm time (**k**), and percentage of open/total-arm entries (**l**). Data are presented as the mean ± SEM, *n* = 10; ns, no statistical significance; ^**^p < 0.05, ^**^p < 0.01, and ^***^p < 0.001, vs. Con

As depicted in Fig. 4, anxiety-like behavior was tested in animals by the OFT and EPM. When exposed to the OFT, HT mice made significantly fewer entries into and spent less time in the central zone than did control mice (Fig. 4b, c), while motor function examined by total distance and mean speed did not differ between groups (Fig. 4d, e). In the EPM, HT mice tended to spend less time in and made fewer entries into the open arms relative to control animals (Fig. 4i, j). Additionally, HT mice exhibited a significant decrement in the percentage of open-arm time and number of open-arm entries (Fig. 4k, l).

### Euthyroid HT induces depressive-like behavior in mice

**Fig. 5:**
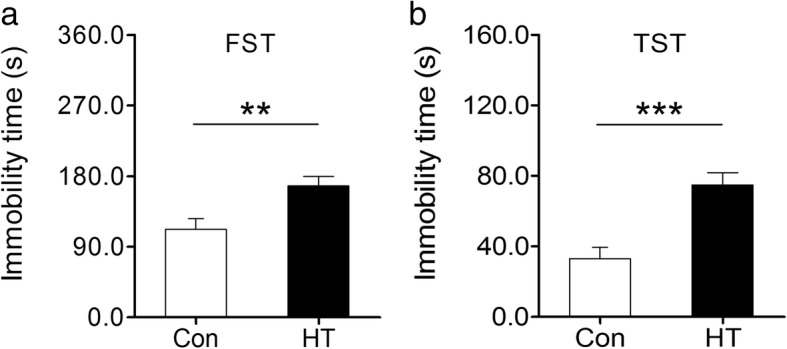
Euthyroid HT induces depressive-like behavior in mice. The FST and TST were performed to assess depressive-like states in animals. **a** Immobility time in the FST. **b** Immobility time in the TST. Data are presented as the mean ± SEM, *n* = 10; ^**^p < 0.01, and ^***^p < 0.001, vs. Con

As depicted in Fig. 5, depressive-like behavior was evaluated in animals by the FST and TST. In the FST, HT mice showed a significant elevation in immobility time compared to control mice (Fig. 5a). This behavioral pattern was also detected in the TST, with HT mice spending more time in immobility state (Fig. 5b). Consistent with these results, rearing and grooming actions as assessed during the OFT were markedly reduced in HT mice compared to those in control mice (Fig. 4f, g), suggestive of a depressive-like state.

### Euthyroid HT induces microglia and astroglia activation in the frontal cortex

**Fig. 6:**
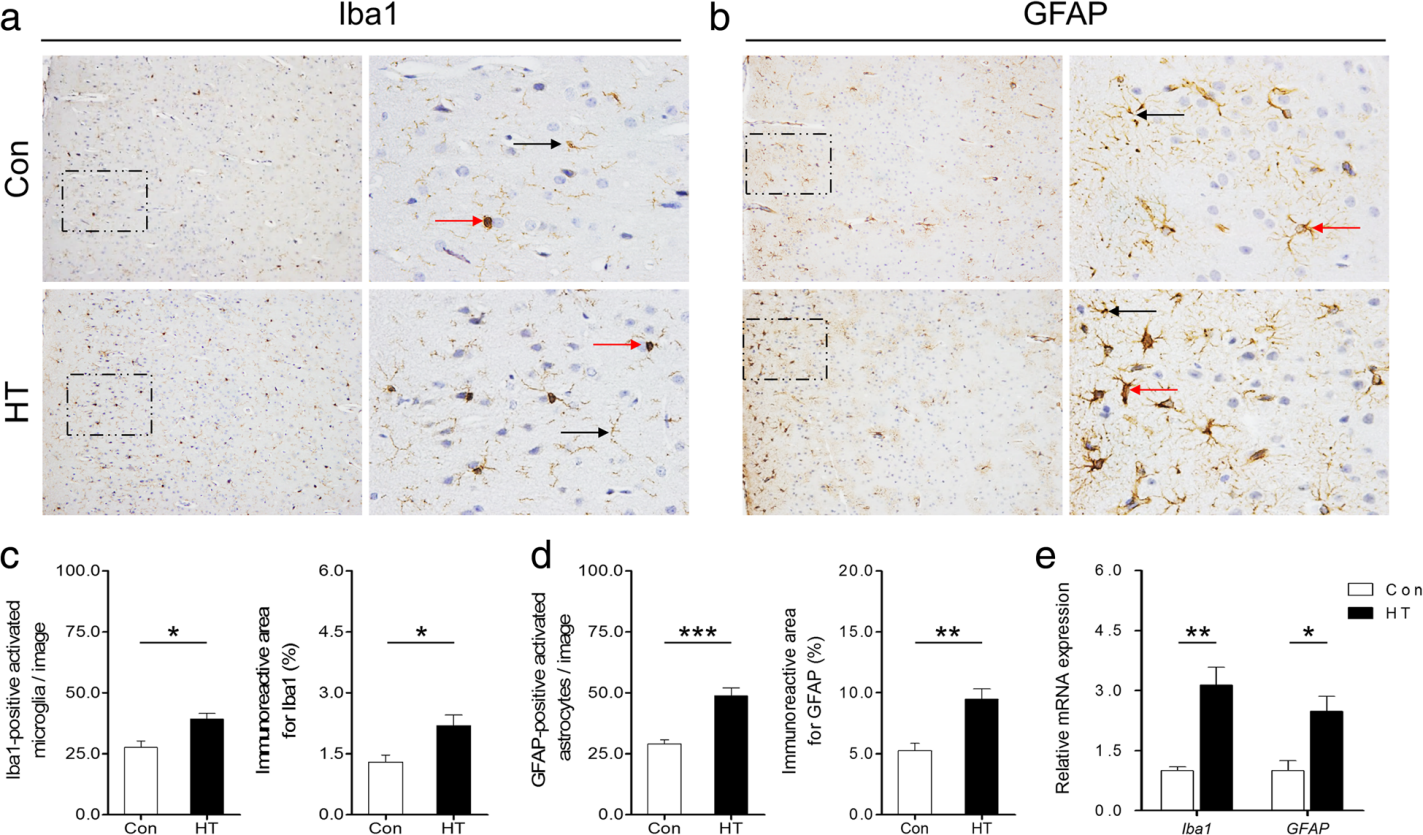
Euthyroid HT induces microglia and astroglia activation in the frontal cortex. IHC and real-time RT-PCR were performed to assess the activation state of microglia and astrocytes in animals. **a**, **b** Representative images of Iba1 (microglia marker) and GFAP (astrocyte marker) staining in the frontal cortex. Each right-hand panel (× 400) depicts a magnified image of the boxed area of the corresponding image in the left panel (× 100). Black arrows indicate resting microglia or astroglia. Red arrows indicate activated microglia or astroglia. **c** Quantitative analysis of Iba1 immunoreactivity. **d** Quantitative analysis of GFAP immunoreactivity. **e** PCR analysis of *Iba1* and *GFAP*. Data are presented as the mean ± SEM, *n* = 5; ^*^p < 0.05, ^**^p < 0.01, and ^***^p < 0.001, vs. Con

Microglia and astroglia are the main executors in the process of neuroinflammation [20]. Therefore, we examined the activation status of microglia and astrocytes in the frontal cortex of mice from both groups by using immunohistochemistry, real-time RT-PCR, and TEM. Representative images of Iba-1 (for microglia) and GFAP (for astrocytes) staining are shown in Fig. 6a, b. We noted that there was an apparent increase in microglial activation in HT mice compared to that in controls, as demonstrated by the greater numbers of activated microglia and higher percentages of Iba1-stained areas in the captured photographs (Fig. 6c). Similarly, the astrocytes also showed intense activation, identified by more activated cells and greater areas of GFAP expression in HT mice than in controls (Fig. 6d). These immunohistochemical results demonstrated that euthyroid HT induced microglia and astroglia activation in the frontal cortex. A quantitative analysis of *Iba1* and *GFAP* mRNA levels in the frontal cortex (Fig. 6e) confirmed these results.

**Fig. 7:**
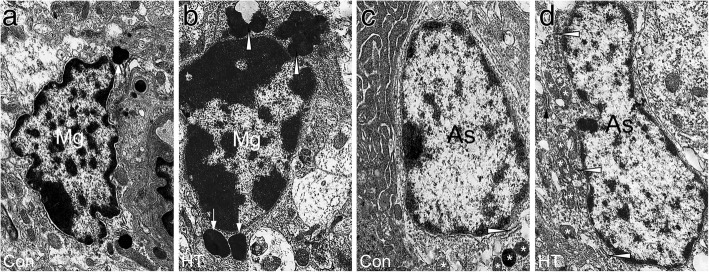
Ultrastructural observations of microglia and astrocytes in the frontal cortex. **a**, **b** Representative TEM images of microglia. The control group (**a**) showed an irregular nucleolus with chromatin condensation beneath the nuclear membrane and a primary lysosome (arrow). In contrast, the HT group (**b**) showed an enlarged nucleolus with increased lysosomes including primary lysosomes (arrows) and secondary lysosomes (arrowheads). Mg, microglia. Magnification × 12,000**. c**, **d** Representative TEM images of astrocytes. The control group (**c**) showed the classical appearance of astrocyte with a narrow rim of chromatin beneath the nuclear membrane. The cytoplasm was pale, contained an endoplasmic reticulum (arrowheads) and a few mitochondria (asterisk). In contrast, the astroglia in the HT group (**d**) had an organelle-rich cytoplasm that included a well-developed Golgi apparatus (arrow), some endoplasmic reticulum (arrowheads), and many mitochondria (asterisks). As, astroglia. Magnification × 10,000

We also observed the ultrastructural features of glial cells by using TEM (Fig. 7). In HT mice, an intense microglial reaction was observed in the frontal cortex. The activated microglia displayed an enlarged nucleus with large, dense clumps of heterochromatin beneath the nuclear envelope. The cytoplasm of glial cells from HT mice contained more lysosomes, including primary lysosomes and secondary lysosomes, than that of control mice (Fig. 7b). These pathological features were associated with phagocytic cells [41]. In addition, the astrocytes, identified by their classical appearance of a narrow rim of chromatin beneath the nuclear membrane, also showed signs of activation in HT mice. These cells tended to show the features involved in active proteosynthesis [42], such as a well-developed Golgi apparatus, some endoplasmic reticulum, and many mitochondria, in HT mice (Fig. 7d).

### Euthyroid HT promotes proinflammatory cytokine expression in the frontal cortex

**Fig. 8:**
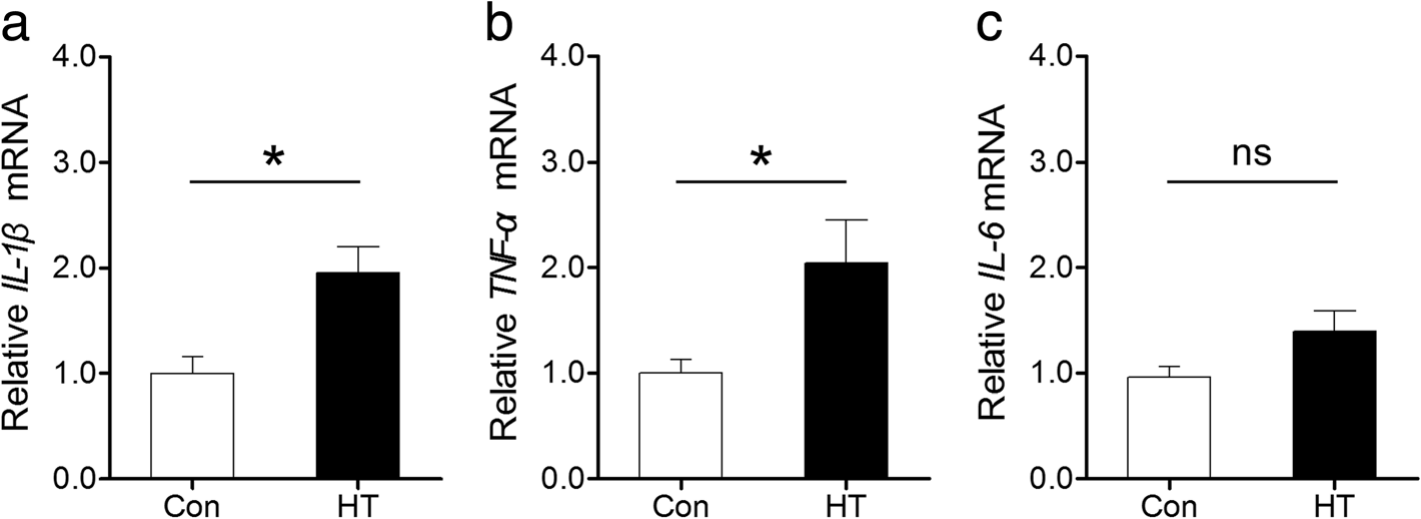
Euthyroid HT promotes proinflammatory cytokine expression in the frontal cortex. Proinflammatory cytokine expression was probed by real-time RT-PCR. **a**–**c** Expression of *IL-1β* (**a**), *TNF-α* (**b**), and *IL-6* (**c**) mRNA. Data are presented as the mean ± SEM, *n* = 5; ns, no statistical significance; ^*^p < 0.05, vs. Con

Activated neuroglia are widely accepted to be able to produce classical proinflammatory cytokines, such as IL-1β, TNF-α, and IL-6 [19]. Accordingly, we probed expression of the cytokines by real-time RT-PCR. Here, frontal cortex expression of *IL-1β* and *TNF-α* was significantly upregulated in HT mice compared to the expression in controls (Fig. 8a, b). In line with this, there was a tendency (albeit not significant) for higher *IL-6* expression in the frontal cortex of HT mice than in that of controls (*p* = 0.08, Fig. 8c).

### Euthyroid HT does not induce neuronal apoptosis in the frontal cortex

**Fig. 9:**
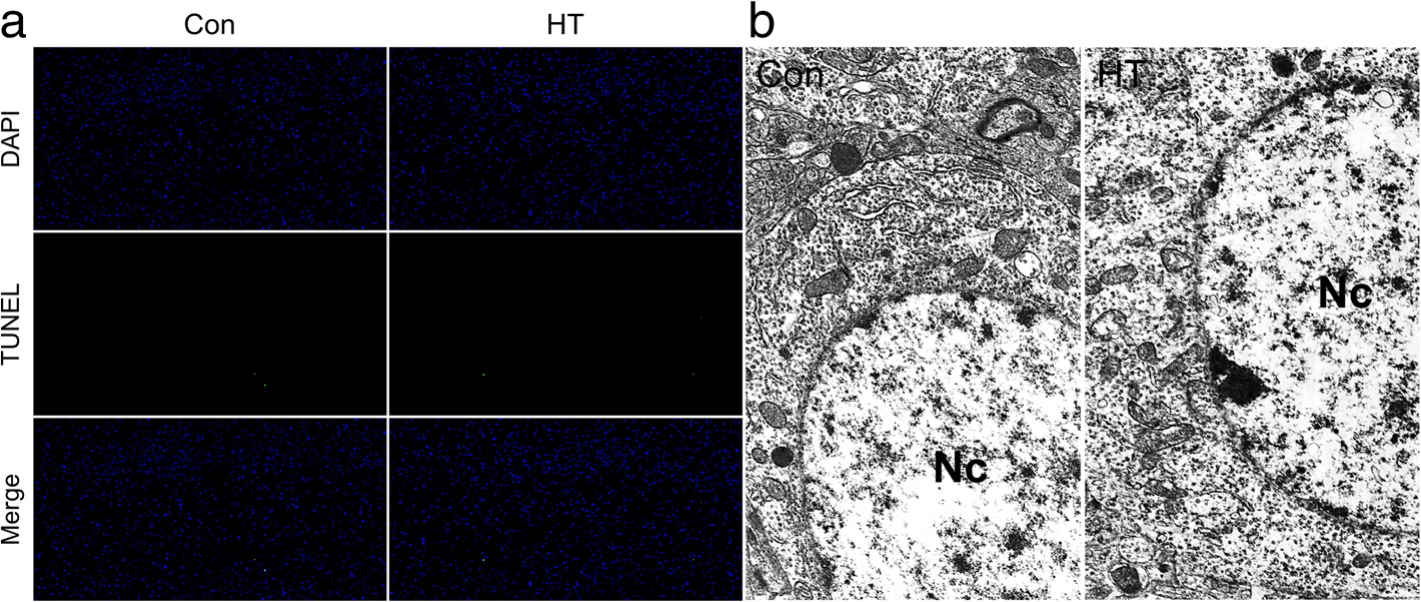
Euthyroid HT does not induce neuronal apoptosis in the frontal cortex. Neuronal apoptosis was identified by the TUNEL staining and TEM. **a** Representative fluorescent images showing TUNEL-labeled (green) apoptotic cells counterstained with DAPI (blue) in the frontal cortex of control mice and HT mice (*n* = 5/group). Magnification × 100. **b** Representative TEM images of frontal cortex neurons in Con mice and HT mice (*n* = 3/group). The neuronal nuclei (Nc) had no signs of apoptosis, such as chromatin margination or nuclear condensation, in the neurons examined. Magnification × 8000

In this study, we performed TUNEL staining and TEM to identify neuronal apoptosis in the frontal cortex. As shown in Fig. 9a, we did not observe any changes in TUNEL-positive neurons in the frontal cortex between groups. In addition, ultrastructure of frontal cortex neurons in HT mice was similar to Con mice with the nucleus with intact nuclear membranes and evenly distributed chromatin (Fig. 9b). There were no apoptotic features, such as chromatin margination or nuclear condensation [43], in the neurons examined.

### Euthyroid HT alters 5-HT signaling in the frontal cortex

**Fig. 10:**
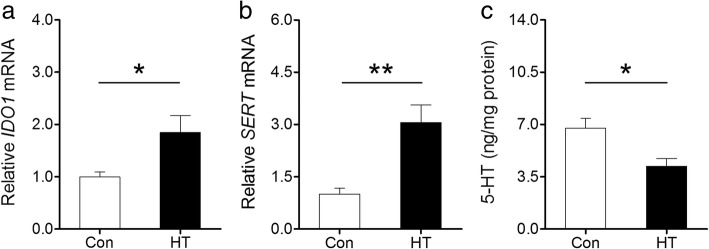
Euthyroid HT alters 5-HT signaling in the frontal cortex. **a**, **b** Expression of *IDO1* and *SERT* mRNA (*n* = 5). **c** 5-HT concentrations (*n* = 7). Data are presented as the mean ± SEM; ^*^p < 0.05, and ^**^p < 0.01, vs. Con

Since our data clearly showed inflammation induction in the frontal cortex of HT mice, we further extended the work to study key mediators involved in the central 5-HT signaling that were sensitive to inflammatory cytokines [23]. We found that mRNA expression of tryptophan-degrading enzyme indoleamine-2,3-dioxygenase (IDO1) and 5-HT transporter (SERT) was significantly higher in HT mice than in control mice (Fig. 10a, b). As expected, the levels of 5-HT measured by ELISA showed a significant reduction in the frontal cortex of HT mice compared to that in controls (Fig. 10c).

## Discussion

Tg is a well-known thyroid auto-antigen associated with the development of thyroiditis in both rodents and humans. Tg-induced thyroiditis is a classic model for studying HT [24] and can be established in susceptible mice, such as NOD mice, leading to the development of HT-like illness characterized by monocyte infiltration into the thyroid gland and presence of autoantibodies against Tg and TPO, which are serological hallmarks of HT [2]. Thus, this model has been widely used to explore the pathogenesis of HT and test therapeutics [24, 44, 45]. In our study, mice immunized with Tg showed intrathyroidal monocyte infiltration and rising serum thyroid autoantibody (TA) levels accompanied by normal T3, T4, and TSH levels, which defines euthyroid HT in humans. To our knowledge, our study is the first to use this model to investigate the effect of HT itself on emotional function in mice, focusing on the possible contribution of neuroinflammation in mediating such an effect.

Thyroid diseases resulting in thyroid dysfunctions are well known to be able to induce psychological deficits, including anxiety and depression [4]. However, the literature on the emotional effects of euthyroid HT are limited and controversial. An early epidemiological study indicated that no association was found between thyroid autoantibodies and anxiety or depression, neither crude nor adjusted for T4 and TSH [46]. On the other hand, other researchers reported impairments of mental well-being in patients with HT that were shown to be independent of thyroid function [5–8]. In this study, we first investigated whether HT affected emotional performance regardless of thyroid dysfunctions in mice. Here, HT mice displayed more anxiety-like behavior during the OFT and EPM than control mice despite a similar thyroid functional state between groups. These mice also displayed prolonged immobility time in the FST and TST, suggestive of depressive-like behavior. These results indicated that HT itself induced behaviors relevant to mood disorders in mice, providing preliminary evidence to support the clinical literature linking euthyroid HT to increased emotional reactivity.

Recently, the crucial role of neuroinflammation in the development of psychological disorders including depression and anxiety has received more attention. Characteristic features of neuroinflammation include the activation of microglia and astroglia and generation of proinflammatory cytokines [19]. An increasing number of human studies have reported microglia activation in the CNS of patients with psychiatric disorders, such as depression, schizophrenia, and anxiety [47, 48]. Results from animal models have shown that neuroinflammation is closely associated with abnormal emotional behavior, which could be improved after antiinflammatory treatment [49–51]. Notably, studies on brain biopsy revealed activated microglia and reactive astrocytes in patients with Hashimoto’s encephalopathy [13, 14], a severe form of a deterioration of the CNS in patients suffering with HT. In the present study, the effects of HT itself on neuroinflammation were examined. Using immunohistochemistry, we found that microglia and astrocytes were activated in the frontal cortex of HT mice. Ultrastructural observations further confirmed glia activation in HT mice, with microglia showing phagocytic function and astrocytes tending to show features involved in active proteosynthesis. Moreover, these observations were accompanied by an increase in the expression of glial marker *Iba1* and *GFAP* as well as pro-inflammatory cytokines *IL-1β* and *TNF-α.* These results demonstrated that HT induced neuroinflammation in the euthyroid state. Thus, HT-induced emotional alterations may be, at least partially, attributed to neuroinflammation.

The mechanisms by which HT induces neuroinflammation in the euthyroid state are still unknown. A recent study showed experimental hyperthyroidism promoted activation of microglia and astrocytes in the cerebral cortex of young mice [52]. In another animal model, hypothyroidism was associated with an increased number of astrocyte cells in the brain [53]. In our study, neuroinflammatory reactions, such as glia activation, were unlikely to be due to thyroid dysfunctions, because serum T3 and T4, as well as TSH, were within the normal range. On the other hand, increased TA themselves may also be pathogenic, given that anti-TPO specifically binds to astrocytes in vitro [54]. Moreover, different studies described the presence of antigenic sites for TA on neural tissues [55, 56], and most recently, anti-Tg has been shown to immunolocalize to vascular smooth muscle of all limbic regions, including frontal cortex [57]. The present study described the increased presence of TA in the frontal cortex of HT mice. It appears reasonable to then propose that TA may cross-react with auto-antigens expressed in the brain and modulate local immune responses. Further studies are needed to explore the detailed mechanisms underlying neuroinflammation in the context of euthyroid HT.

Interestingly, despite this inflammatory response, no signs of neuronal apoptosis were visible by the TUNEL staining and TEM in the frontal cortex of HT mice. This result was in line with previous studies showing that increased brain inflammatory response was not functionally associated to neuronal apoptosis or neuronal damage [58–61]. It is interesting to speculate that HT could not induce neuronal damage. This may explain the very consistent finding of normal neural activity on resting-state functional magnetic resonance imaging in patients with euthyroid HT [62]. Utilization of other markers to assess neurodegeneration or neurogenesis would have been useful but it was beyond the scope of the study. Despite no evident structural alterations of neurons, emotional dysfunction can occur due to functional impairments, for example, of the serotonergic signaling system, which are important mechanisms in the regulation of mood [63].

Recent studies have revealed that the serotonergic signaling system may be a major target for immune mediators, such as proinflammatory cytokines. In vitro results have shown that treatment with proinflammatory cytokines, such as IL-1β and TNF-α, directly induce IDO1 and SERT expression in neuroglia and/or neurons [64–67]. In vivo studies have suggested that central inflammatory signaling activation significantly upregulates IDO1 and/or SERT, resulting in depressive-like behavior in animals [68–70]. Indeed, IDO-1 and SERT are crucial regulators of central 5-HT signaling, which plays vital roles in the development of psychiatric symptoms in humans and animals [63]. In this study, frontal cortex expression of *IDO-1* and *SERT* was sharply increased in HT mice. Moreover, the 5-HT concentration in the frontal cortex was lower in HT mice than in controls. These results demonstrated that elevated expression of IL-1β and TNF-α paralleled by glial activation provoked by HT may impact 5-HT signaling and thereby contribute to the emotional disturbance. Different studies have revealed that antiinflammatory drugs such as celecoxib have an inhibitory effect on the neuroinflammatory response [38, 71, 72] and that celecoxib therapy exerts a beneficial effect on psychiatric symptoms in some cases [73, 74]. Detailed data on the effects of antiinflammatory therapy upon psychiatric symptoms related with euthyroid HT is of interest for future studies.

There are some deficiencies in the present study. First, all behavioral tests were conducted on the same group of mice. The stress associated with exposure of the mice to behavioral tests may consequently affect performance in the subsequent behavioral tests; therefore, we are unable to entirely exclude an interactive effect among the behavioral tests. However, the mice in control group underwent the same behavioral tests in this study. Second, although IL-1β, TNF-α, and IL-6 are the most common cytokines involved in process of neuroinflammation, they do not represent the entire range of inflammatory molecules. Therefore, other inflammatory cytokines related to neuroinflammation remain to be considered. Third, this study focused on the frontal cortex because this brain area is involved in mood control and is the primary region affected in euthyroid HT patients. Other brain regions relevant to mood regulation, such as the hippocampus, should be examined in further studies.

## Conclusions

In summary, emerging clinical studies have shown that HT renders individuals vulnerable to psychopathology in the euthyroid state. Using an animal model, this study provides further evidence for such arguments, showing for the first time that euthyroid HT induced emotional alterations in mice, at least partially, through induction of neuroinflammation and alterations in 5-HT signaling in the frontal cortex. These findings provide preliminary leads to further explore the potential role of neuroinflammation in mediating these psychological consequences of euthyroid HT and to develop effective approaches to combat them.

## Abbreviations

5-HT: Serotonin
Anti-Tg: Anti-thyroglobulin antibody
Anti-TPO: Anti-thyroid peroxidase antibody
CFA: Complete Freund’s adjuvant
ECLIA: Electrochemiluminescence immunoassay
ELISA: Enzyme-linked immunosorbent assay
EPM: Elevated plus maze
FST: Forced swimming test
GFAP: Glial fibrillary acidic protein
HE: Hematoxylin and eosin
HT: Hashimoto’s thyroiditis
Iba1: Ionized calcium-binding adapter molecule 1
IDO1: Indoleamine-2,3-dioxygenase
IFA: Incomplete Freund’s adjuvant
IL-1β: Interleukin-1β
IL-6: Interleukin-6
OFT: Open field test
SERT: Serotonin transporter
T3: Triiodothyronine
T4: Thyroxin
TEM: Transmission electron microscopy
Tg: Thyroglobulin
TNF-α: Tumor necrosis factor-α
TSH: Thyroid-stimulating hormone
TST: Tail suspension test
TUNEL: Terminal deoxynucleotidyl transferase-mediated dUTP-biotin nick end labeling

## References

[1] Siriweera Eranga Himalee, Ratnatunga Neelakanthi Vajira Illangakoon (2010). Profile of Hashimoto's Thyroiditis in Sri Lankans: Is There an Increased Risk of Ancillary Pathologies in Hashimoto's Thyroiditis?. Journal of Thyroid Research.

[2] Pearce EN, Farwell AP, Braverman LE (2003). Thyroiditis. N Engl J Med.

[3] Kapila K, Sathar SA, Al-Rabah NA, Prahash A, Seshadri MS (1995). Chronic lymphocytic (Hashimoto’s) thyroiditis in Kuwait diagnosed by fine needle aspirates. Ann Saudi Med.

[4] Bauer M, Goetz T, Glenn T, Whybrow PC (2008). The thyroid-brain interaction in thyroid disorders and mood disorders. J Neuroendocrinol.

[5] Carta MG, Hardoy MC, Carpiniello B, Murru A, Marci AR, Carbone F, Deiana L, Cadeddu M, Mariotti S (2005). A case control study on psychiatric disorders in Hashimoto disease and Euthyroid goitre: not only depressive but also anxiety disorders are associated with thyroid autoimmunity. Clin Pract Epidemiol Ment Health.

[6] Kirim S, Keskek SO, Koksal F, Haydardedeoglu FE, Bozkirli E, Toledano Y (2012). Depression in patients with euthyroid chronic autoimmune thyroiditis. Endocr J.

[7] Giynas Ayhan M, Uguz F, Askin R, Gonen MS (2014). The prevalence of depression and anxiety disorders in patients with euthyroid Hashimoto's thyroiditis: a comparative study. Gen Hosp Psychiatry.

[8] Yalcin MM, Altinova AE, Cavnar B, Bolayir B, Akturk M, Arslan E, Ozkan C, Cakir N, Balos Toruner F (2017). Is thyroid autoimmunity itself associated with psychological well-being in euthyroid Hashimoto's thyroiditis?. Endocr J.

[9] Mussig K, Kunle A, Sauberlich AL, Weinert C, Ethofer T, Saur R, Klein R, Haring HU, Klingberg S, Gallwitz B, Leyhe T (2012). Thyroid peroxidase antibody positivity is associated with symptomatic distress in patients with Hashimoto's thyroiditis. Brain Behav Immun.

[10] Piga M, Serra A, Deiana L, Loi GL, Satta L, Di Liberto M, Mariotti S (2004). Brain perfusion abnormalities in patients with euthyroid autoimmune thyroiditis. Eur J Nucl Med Mol Imaging.

[11] Millan MJ, Rivet JM, Gobert A (2016). The frontal cortex as a network hub controlling mood and cognition: probing its neurochemical substrates for improved therapy of psychiatric and neurological disorders. J Psychopharmacol.

[12] Leyhe T, Ethofer T, Bretscher J, Kunle A, Sauberlich AL, Klein R, Gallwitz B, Haring HU, Fallgatter A, Klingberg S (2013). Low performance in attention testing is associated with reduced grey matter density of the left inferior frontal gyrus in euthyroid patients with Hashimoto’s thyroiditis. Brain Behav Immun.

[13] Doherty CP, Schlossmacher M, Torres N, Bromfield E, Samuels MA, Folkerth R (2002). Hashimoto’s encephalopathy mimicking Creutzfeldt-Jakob disease: brain biopsy findings. J Neurol Neurosurg Psychiatry.

[14] Zhao W, Li J, Wang J, Guo Y, Tuo H, Kang Z, Jiang B, Wang R, Wang D (2011). A case of Hashimoto encephalopathy: clinical manifestation, imaging, pathology, treatment, and prognosis. Neurologist.

[15] Cardenas-Roldan J, Rojas-Villarraga A, Anaya JM (2013). How do autoimmune diseases cluster in families? A systematic review and meta-analysis. BMC Med.

[16] Bruce TO (2008). Comorbid depression in rheumatoid arthritis: pathophysiology and clinical implications. Curr Psychiatry Rep.

[17] Jeltsch-David H, Muller S (2016). Autoimmunity, neuroinflammation, pathogen load: a decisive crosstalk in neuropsychiatric SLE. J Autoimmun.

[18] Fuggle NR, Howe FA, Allen RL, Sofat N (2014). New insights into the impact of neuro-inflammation in rheumatoid arthritis. Front Neurosci.

[19] Kempuraj D, Thangavel R, Selvakumar GP, Zaheer S, Ahmed ME, Raikwar SP, Zahoor H, Saeed D, Natteru PA, Iyer S, Zaheer A (2017). Brain and peripheral atypical inflammatory mediators potentiate Neuroinflammation and neurodegeneration. Front Cell Neurosci.

[20] Hendriksen E, van Bergeijk D, Oosting RS, Redegeld FA (2017). Mast cells in neuroinflammation and brain disorders. Neurosci Biobehav Rev.

[21] Capuron L, Miller AH (2011). Immune system to brain signaling: neuropsychopharmacological implications. Pharmacol Ther.

[22] Najjar S, Pearlman DM, Alper K, Najjar A, Devinsky O (2013). Neuroinflammation and psychiatric illness. J Neuroinflammation.

[23] Rosenblat JD, Cha DS, Mansur RB, McIntyre RS (2014). Inflamed moods: a review of the interactions between inflammation and mood disorders. Prog Neuro-Psychopharmacol Biol Psychiatry.

[24] Damotte D, Colomb E, Cailleau C, Brousse N, Charreire J, Carnaud C (1997). Analysis of susceptibility of NOD mice to spontaneous and experimentally induced thyroiditis. Eur J Immunol.

[25] Sadelain MW, Qin HY, Lauzon J, Singh B (1990). Prevention of type I diabetes in NOD mice by adjuvant immunotherapy. Diabetes.

[26] Qin HY, Sadelain MW, Hitchon C, Lauzon J, Singh B (1993). Complete Freund’s adjuvant-induced T cells prevent the development and adoptive transfer of diabetes in nonobese diabetic mice. J Immunol.

[27] Lee IF, Qin H, Trudeau J, Dutz J, Tan R (2004). Regulation of autoimmune diabetes by complete Freund’s adjuvant is mediated by NK cells. J Immunol.

[28] Chen Z, Xu YY, Wu R, Han YX, Yu Y, Ge JF, Chen FH. Impaired learning and memory in rats induced by a high-fat diet: involvement with the imbalance of nesfatin-1 abundance and copine 6 expression. J Neuroendocrinol. 2017. 10.1111/jne.12462. 10.1111/jne.12462 28211103

[29] Ge JF, Gao WC, Cheng WM, Lu WL, Tang J, Peng L, Li N, Chen FH (2014). Orcinol glucoside produces antidepressant effects by blocking the behavioural and neuronal deficits caused by chronic stress. Eur Neuropsychopharmacol.

[30] Li M, Li C, Yu H, Cai X, Shen X, Sun X, Wang J, Zhang Y, Wang C (2017). Lentivirus-mediated interleukin-1beta (IL-1beta) knock-down in the hippocampus alleviates lipopolysaccharide (LPS)-induced memory deficits and anxiety- and depression-like behaviors in mice. J Neuroinflammation.

[31] Yu Z, Han Y, Shen R, Huang K, Xu YY, Wang QN, Zhou SS, Xu DX, Tao FB (2018). Gestational di-(2-ethylhexyl) phthalate exposure causes fetal intrauterine growth restriction through disturbing placental thyroid hormone receptor signaling. Toxicol Lett.

[32] Wang X, Liu H, Zhang Y, Li J, Teng X, Liu A, Yu X, Shan Z, Teng W (2015). Effects of isolated positive maternal thyroglobulin antibodies on brain development of offspring in an experimental autoimmune thyroiditis model. Thyroid.

[33] Houlden A, Goldrick M, Brough D, Vizi ES, Lenart N, Martinecz B, Roberts IS, Denes A (2016). Brain injury induces specific changes in the caecal microbiota of mice via altered autonomic activity and mucoprotein production. Brain Behav Immun.

[34] Paxinos G, Franklin K (2013). Paxinos and Franklin's the mouse brain in stereotaxic coordinates.

[35] Kreutzberg GW (1996). Microglia: a sensor for pathological events in the CNS. Trends Neurosci.

[36] Sofroniew MV, Vinters HV (2010). Astrocytes: biology and pathology. Acta Neuropathol.

[37] Wixey JA, Reinebrant HE, Spencer SJ, Buller KM (2011). Efficacy of post-insult minocycline administration to alter long-term hypoxia-ischemia-induced damage to the serotonergic system in the immature rat brain. Neuroscience.

[38] Kaizaki A, Tien LT, Pang Y, Cai Z, Tanaka S, Numazawa S, Bhatt AJ, Fan LW (2013). Celecoxib reduces brain dopaminergic neuronal dysfunction, and improves sensorimotor behavioral performance in neonatal rats exposed to systemic lipopolysaccharide. J Neuroinflammation.

[39] Yanguas-Casas N, Barreda-Manso MA, Nieto-Sampedro M, Romero-Ramirez L (2014). Tauroursodeoxycholic acid reduces glial cell activation in an animal model of acute neuroinflammation. J Neuroinflammation.

[40] Wang F, Wu Z, Zha X, Cai Y, Wu B, Jia X, Zhu D (2017). Concurrent administration of thyroxine and donepezil induces plastic changes in the prefrontal cortex of adult hypothyroid rats. Mol Med Rep.

[41] Dahlke C, Saberi D, Ott B, Brand-Saberi B, Schmitt-John T, Theiss C (2015). Inflammation and neuronal death in the motor cortex of the wobbler mouse, an ALS animal model. J Neuroinflammation.

[42] Casamenti F, Prosperi C, Scali C, Giovannelli L, Colivicchi MA, Faussone-Pellegrini MS, Pepeu G (1999). Interleukin-1beta activates forebrain glial cells and increases nitric oxide production and cortical glutamate and GABA release in vivo: implications for Alzheimer’s disease. Neuroscience.

[43] Cheng C, Zochodne DW (2003). Sensory neurons with activated caspase-3 survive long-term experimental diabetes. Diabetes.

[44] Jin Z, Mori K, Fujimori K, Hoshikawa S, Tani J, Satoh J, Ito S, Satomi S, Yoshida K (2004). Experimental autoimmune thyroiditis in nonobese diabetic mice lacking interferon regulatory factor-1. Clin Immunol.

[45] Mori K, Yoshida K, Tani J, Nakagawa Y, Hoshikawa S, Ozaki H, Ito S (2008). Effects of angiotensin II blockade on the development of autoimmune thyroiditis in nonobese diabetic mice. Clin Immunol.

[46] Engum A, Bjoro T, Mykletun A, Dahl AA (2005). Thyroid autoimmunity, depression and anxiety; are there any connections? An epidemiological study of a large population. J Psychosom Res.

[47] van der Doef TF, Doorduin J, van Berckel BNM, Cervenka S (2015). Assessing brain immune activation in psychiatric disorders: clinical and preclinical PET imaging studies of the 18-kDa translocator protein. Clin Transl Imaging.

[48] Setiawan E, Wilson AA, Mizrahi R, Rusjan PM, Miler L, Rajkowska G, Suridjan I, Kennedy JL, Rekkas PV, Houle S, Meyer JH (2015). Role of translocator protein density, a marker of neuroinflammation, in the brain during major depressive episodes. JAMA Psychiatry.

[49] Wang YL, Han QQ, Gong WQ, Pan DH, Wang LZ, Hu W, Yang M, Li B, Yu J, Liu Q (2018). Microglial activation mediates chronic mild stress-induced depressive- and anxiety-like behavior in adult rats. J Neuroinflammation.

[50] da Silva Dias IC, Carabelli B, Ishii DK, de Morais H, de Carvalho MC, Rizzo de Souza LE, Zanata SM, Brandao ML, Cunha TM, Ferraz AC (2016). Indoleamine-2,3-dioxygenase/kynurenine pathway as a potential pharmacological target to treat depression associated with diabetes. Mol Neurobiol.

[51] Haile M, Boutajangout A, Chung K, Chan J, Stolper T, Vincent N, Batchan M, D'Urso J, Lin Y, Kline R, et al. The Cox-2 Inhibitor Meloxicam Ameliorates Neuroinflammation and Depressive Behavior in Adult Mice after Splenectomy. J Neurophysiol Neurol Disord. 2016;3:1–9. PMC5380921 28393111

[52] Noda M (2018). Thyroid hormone in the CNS: contribution of neuron-glia interaction. Vitam Horm.

[53] Cortes C, Eugenin E, Aliaga E, Carreno LJ, Bueno SM, Gonzalez PA, Gayol S, Naranjo D, Noches V, Marassi MP (2012). Hypothyroidism in the adult rat causes incremental changes in brain-derived neurotrophic factor, neuronal and astrocyte apoptosis, gliosis, and deterioration of postsynaptic density. Thyroid.

[54] Blanchin S, Coffin C, Viader F, Ruf J, Carayon P, Potier F, Portier E, Comby E, Allouche S, Ollivier Y (2007). Anti-thyroperoxidase antibodies from patients with Hashimoto’s encephalopathy bind to cerebellar astrocytes. J Neuroimmunol.

[55] Ota K, Matsui M, Milford EL, Mackin GA, Weiner HL, Hafler DA (1990). T-cell recognition of an immunodominant myelin basic protein epitope in multiple sclerosis. Nature.

[56] Moodley K, Botha J, Raidoo DM, Naidoo S (2011). Immuno-localisation of anti-thyroid antibodies in adult human cerebral cortex. J Neurol Sci.

[57] Naicker M, Naidoo S (2018). Expression of thyroid-stimulating hormone receptors and thyroglobulin in limbic regions in the adult human brain. Metab Brain Dis.

[58] Mouihate A, Pittman QJ (1998). Lipopolysaccharide-induced fever is dissociated from apoptotic cell death in the rat brain. Brain Res.

[59] Aid S, Langenbach R, Bosetti F (2008). Neuroinflammatory response to lipopolysaccharide is exacerbated in mice genetically deficient in cyclooxygenase-2. J Neuroinflammation.

[60] Francois A, Terro F, Quellard N, Fernandez B, Chassaing D, Janet T, Rioux Bilan A, Paccalin M, Page G (2014). Impairment of autophagy in the central nervous system during lipopolysaccharide-induced inflammatory stress in mice. Mol Brain.

[61] Sapin E, Peyron C, Roche F, Gay N, Carcenac C, Savasta M, Levy P, Dematteis M (2015). Chronic intermittent hypoxia induces chronic low-grade neuroinflammation in the dorsal hippocampus of mice. Sleep.

[62] Quinque EM, Karger S, Arelin K, Schroeter ML, Kratzsch J, Villringer A (2014). Structural and functional MRI study of the brain, cognition and mood in long-term adequately treated Hashimoto's thyroiditis. Psychoneuroendocrinology.

[63] Cowen PJ (2008). Serotonin and depression: pathophysiological mechanism or marketing myth?. Trends Pharmacol Sci.

[64] Hochstrasser T, Ullrich C, Sperner-Unterweger B, Humpel C (2011). Inflammatory stimuli reduce survival of serotonergic neurons and induce neuronal expression of indoleamine 2,3-dioxygenase in rat dorsal raphe nucleus organotypic brain slices. Neuroscience.

[65] Zunszain PA, Anacker C, Cattaneo A, Choudhury S, Musaelyan K, Myint AM, Thuret S, Price J, Pariante CM (2012). Interleukin-1beta: a new regulator of the kynurenine pathway affecting human hippocampal neurogenesis. Neuropsychopharmacology.

[66] Zhu CB, Blakely RD, Hewlett WA. The proinflammatory cytokines interleukin-1beta and tumor necrosis factor-alpha activate serotonin transporters. Neuropsychopharmacology. 2006. 10.1038/sj.npp.1301029. 10.1038/sj.npp.1301029 16452991

[67] Malynn S, Campos-Torres A, Moynagh P, Haase J (2013). The pro-inflammatory cytokine TNF-alpha regulates the activity and expression of the serotonin transporter (SERT) in astrocytes. Neurochem Res.

[68] Fu X, Zunich SM, O'Connor JC, Kavelaars A, Dantzer R, Kelley KW (2010). Central administration of lipopolysaccharide induces depressive-like behavior in vivo and activates brain indoleamine 2,3 dioxygenase in murine organotypic hippocampal slice cultures. J Neuroinflammation.

[69] Zhu CB, Lindler KM, Owens AW, Daws LC, Blakely RD, Hewlett WA (2010). Interleukin-1 receptor activation by systemic lipopolysaccharide induces behavioral despair linked to MAPK regulation of CNS serotonin transporters. Neuropsychopharmacology.

[70] Dobos N, de Vries EF, Kema IP, Patas K, Prins M, Nijholt IM, Dierckx RA, Korf J, den Boer JA, Luiten PG, Eisel UL (2012). The role of indoleamine 2,3-dioxygenase in a mouse model of neuroinflammation-induced depression. J Alzheimers Dis.

[71] Villa V, Thellung S, Corsaro A, Novelli F, Tasso B, Colucci-D'Amato L, Gatta E, Tonelli M, Florio T (2016). Celecoxib inhibits prion protein 90-231-mediated pro-inflammatory responses in microglial cells. Mol Neurobiol.

[72] Mhillaj E, Morgese MG, Tucci P, Furiano A, Luongo L, Bove M, Maione S, Cuomo V, Schiavone S, Trabace L (2018). Celecoxib prevents cognitive impairment and neuroinflammation in soluble amyloid beta-treated rats. Neuroscience.

[73] Muller N, Schwarz MJ, Dehning S, Douhe A, Cerovecki A, Goldstein-Muller B, Spellmann I, Hetzel G, Maino K, Kleindienst N (2006). The cyclooxygenase-2 inhibitor celecoxib has therapeutic effects in major depression: results of a double-blind, randomized, placebo controlled, add-on pilot study to reboxetine. Mol Psychiatry.

[74] Kohler O, Benros ME, Nordentoft M, Farkouh ME, Iyengar RL, Mors O, Krogh J (2014). Effect of anti-inflammatory treatment on depression, depressive symptoms, and adverse effects: a systematic review and meta-analysis of randomized clinical trials. JAMA Psychiatry.

